# An unusual presentation of Boerhaave Syndrome: a case report

**DOI:** 10.4076/1757-1626-2-8000

**Published:** 2009-06-24

**Authors:** Fardod O’Kelly, Kheng Tian Lim, Fiachra Cooke, Narayanasamy Ravi, John Vincent Reynolds

**Affiliations:** Department of Clinical Surgery, Trinity Centre, Trinity College Dublin and St. James’s HospitalDublin 8Ireland

## Abstract

We present a unique case of Boerhaave Syndrome that may highlight the spectrum of barotrauma from a Mallory-Weiss tear to full-thickness perforation. In this case, perforation only became evident following air insufflation at endoscopy.

## Introduction

Boerhaave Syndrome, first described in 1724, is a life threatening consequence of non-iatrogenic rupture of the esophagus, and rupture usually occurs in the left postero-lateral wall of the lower third of the esophagus. The term spontaneous perforation of the esophagus, although commonly used, is an inappropriate term, as the rupture is rarely spontaneous and almost invariably follows barotrauma from a sudden post-emetic rise in esophageal pressure. Although the original report of Boerhaave describes a transverse tear in the lower left esophagus, most tears are longitudinal, varying in length from 0.5 to 20 cm, located on the left postero-lateral wall of the esophagus, 2 to 6 cm above the diaphragm in 80% of cases [1]. The predilection for this site is unclear, but it may result from splaying of muscle fibers and the entrance of blood vessels and nerves at this site. In contrast, Mallory-Weiss tears, first described in 1929, are thought to occur as a result of transient transmural pressure gradients across the esophago-gastric junction as a result of rapid rises in intragastric pressure, typically seen during vomiting and/or retching. As the high pressure gastric system comes into close proximity with the lower pressure system above the lower esophageal sphincter, shearing forces can result in longitudinal lacerations, usually confined to the mucosa.

We report herein a patient that presented with classical Mallory-Weiss features that only presented with pneumomediastinum, subcutaneous emphysema and pneumothorax after endoscopy and air insufflation. The case was managed successfully via a non-operative approach, but may highlight the spectrum of barotrauma between Mallory-Weiss and Boerhaave syndrome.

## Case presentation

A 56-year-old female, Irish, Caucasian patient was transferred from another institution with a 36-hour history of sudden onset dyspepsia and vomiting which was blood-stained. This had awoken her from her sleep and caused her to attend her local emergency department. She had a background history of gastro-esophageal reflux disease but was not on medication. She had undergone no prior surgical procedures.

**Figure 1.**
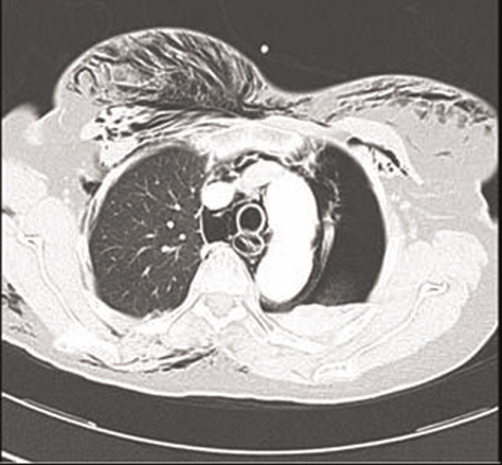
CT study showing extensive subcutaneous emphysema and left-sided pneumothorax, but no demonstration of the esophageal defect.

An esophagogastroduodenoscopy performed prior to her transfer reported marked distal esophagitis, and noted a small “polyp” superior to the esophago-gastric junction. There was no evidence of active bleeding. Following this procedure, the patient became unwell and complained of dyspnoea and neck pain. She also developed significant levels of subcutaneous emphysema tracking across her anterior and posterior chest, her neck bilaterally and into her face, which was more pronounced on the right side. At this point, she was transferred to this centre with a suspected perforation. On arrival she was not systemically unwell, was apyrexic, but had a leucocytosis (13.77 × 10^9^/L) and a neutrophilia (10.46 × 10^9^/L). Her biochemical parameters were otherwise unremarkable. On clinical examination, she exhibited a large left sided pneumothorax in addition to subcutaneous emphysema. She was emergently admitted to the intensive care unit where a 24Fr left intercostal chest tube was inserted and attached to an underwater seal drain following resuscitation in accordance with ATLS® guidelines. A computed tomography (CT) scan of the neck and thorax showed evidence of extensive subcutaneous emphysema within the soft tissues of the chest and mediastinum, and thickening and mixed low attenuation change in the region of the gastric fundus and junction, and air tracking posterior to crus of the diaphragm (Figure 1).

**Figure 2.**
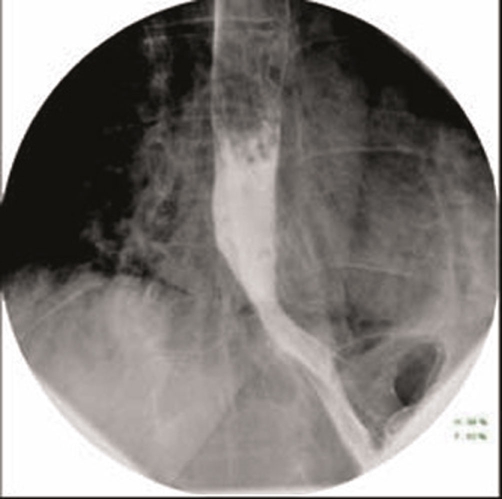
Gastrograffin swallow demonstrating free flow with no extravasation of contrast.

**Figure 3.**
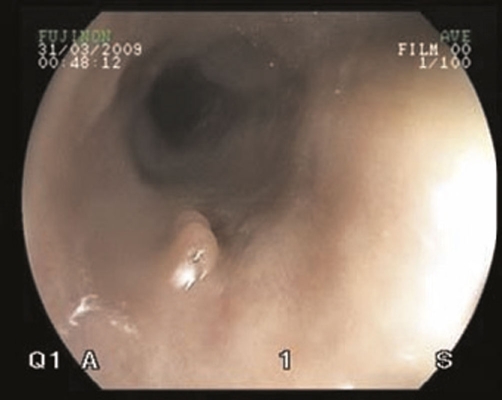
Endoscopy demonstrating region of tear proximal to esophago-gastric junction with no evidence of active haemorrhage.

A gastrograffin swallow was performed and did not identify any esophageal leak despite multiple swallows in erect, supine and prone positions. There was a small hiatus hernia with a small volume of gastro-esophageal reflux, (Figure 2). The patient underwent a barium swallow 24 hours later, and this again showed no evidence of a leak or stricture with a small mucosal irregularity present in the distal esophagus above a small sliding hiatus hernia. An endoscopy was then performed which demonstrated a tear above the gastric cardia, but no evidence of active bleeding (Figure 3).

The patient made an unremarkable recovery following conservative surgical management and was discharged soon after without complication, having made a complete recovery. Patient remains well at six months of follow-up.

## Discussion

This patient presented with what appeared to be a Mallory-Weiss tear initially but manifested as an esophageal perforation following endoscopy. The initial problem may have been a sub-clinical Boerhaave Syndrome, or the initial endoscopy, reportedly atraumatic, may have resulted in a rupture. It may be more likely that a full-thickness tear from barotrauma was opened by air insufflation at the time of endoscopy. There has been speculation as to the site of distal esophageal perforations occurring in a constant region. Korn et al looking at cadaveric specimens, found that insufflation with air caused esophageal rupture at the margin of contact between “clasp” and oblique fibers, and extended upwards, with this connective tissue appearing to constitute a weak point in the distal esophagus near the esophago-gastric junction [2].

To our knowledge this is the first case to highlight this spectrum of barotraumas following vomiting. It is well established that deep Mallory-Weiss tears can lead to the formation of esophageal intramural hematomas [3-6].

## Conclusion

This case report is unique as it, for the first time, demonstrates the transition of a deep Mallory-Weiss tear in the distal esophagus into a Boerhaave syndrome, diagnosed by endoscopic insufflation during gastroscopy. It lends support to the concept that tears in the lower esophagus may all be part of a spectrum of disease which can be assessed using a combination of endoscopic, computed tomographic and contrast studies.
